# Transnasal Endoscopic Approach for the Revision of Rathke’s Cyst and Successful Long-Term Follow-Up: A Case Report

**DOI:** 10.7759/cureus.104987

**Published:** 2026-03-10

**Authors:** Arturo Sotomayor-González, Hiram H Plata-Huerta, Jonathan Ortiz-Rafael, Josefina Alejandra Morales Del Angel, Miriam Delgado Brito

**Affiliations:** 1 Department of Neurological Surgery, Hospital Universitario Dr. José Eleuterio González, Universidad Autónoma de Nuevo León, Monterrey, MEX; 2 Department of Otolaryngology - Head and Neck Surgery, Hospital Universitario Dr. José Eleuterio González, Universidad Autónoma de Nuevo León, Monterrey, MEX; 3 Department of Neurological Surgery, Monterrey Regional Hospital, Institute for Social Security and Services for State Workers, Monterrey, MEX

**Keywords:** endoscopic surgery, marsupialization, rathke's cleft cyst, recurrence, transnasal approach, visual impairment

## Abstract

Rathke's cleft cysts are benign epithelial-lined sellar and suprasellar lesions that are often incidental findings but can cause visual deficits, hypopituitarism, and headaches when they enlarge and compress adjacent structures. Recurrence after surgical treatment remains challenging despite various surgical approaches. Marsupialization has emerged as an alternative technique for complex recurrent cases, establishing permanent communication between the cyst and the nasal cavity while minimizing surgical risk. We present a case of a 76-year-old man with type 2 diabetes mellitus, a history of meningeal tuberculosis, and panhypopituitarism who experienced progressive visual decline following multiple failed Rathke's cleft cyst surgeries. Ophthalmological evaluation revealed right temporal hemianopsia and reduced visual acuity, and magnetic resonance imaging confirmed a recurrent sellar-suprasellar lesion compressing the optic chiasm. Revision endoscopic endonasal surgery was performed using the marsupialization technique, including a type III sphenoidotomy, cyst aperture, dural membrane eversion toward the nasal cavity, and nasoseptal flap placement on the cyst floor. Surgery was completed without complications. At eight-month follow-up, the patient demonstrated complete resolution of hemianopsia, improved visual acuity, and stable postoperative changes without cyst reaccumulation on imaging. Endoscopic transnasal marsupialization represents a safe and effective therapeutic option for recurrent Rathke's cleft cysts, particularly following multiple failed interventions, providing permanent drainage while minimizing manipulation of delicate neurovascular structures. It may also potentially reduce recurrence rates and improve functional outcomes in complex cases.

## Introduction

Rathke’s cleft cysts are benign epithelial-lined sellar and suprasellar lesions, representing 2%-5% of intracranial masses [1]. Rathke's cleft cysts are often incidental, but their enlargement can compress adjacent structures, causing visual deficits, hypopituitarism, and/or headaches [2,3]. Standard treatment includes surgical drainage or excision, which can be performed via a transsphenoidal endoscopic approach. Recurrence remains a challenge, with rates reported between 10% and 33%, and complete excision is not always feasible due to adherence to critical neurovascular structures [4,5]. Marsupialization, which establishes a permanent communication between the cyst and nasal cavity, has emerged as an alternative surgical endoscopic endonasal technique in complex recurrent cases that can provide durable drainage while minimizing surgical risk [6]. Thus, we describe a case presentation with surgical management and postoperative outcome of a patient with a recurrent Rathke’s cleft cyst treated successfully with endoscopic transnasal marsupialization and nasoseptal flap reconstruction. This report was approved by the Ethics and Research Committees of the University Hospital "Dr. José Eleuterio González," and the patient provided verbally informed consent for publication.

## Case presentation

**Figure 1:**
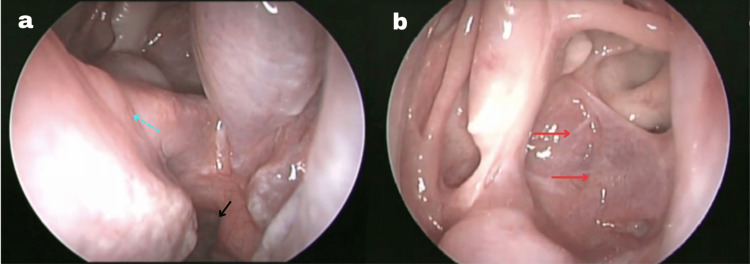
Preoperative endoscopic endonasal view. (a) Endoscopic view of the posterior nasal cavity with surgical changes, past posterior septectomy (blue arrow), nasal choana (black arrow). (b) Endoscopic view of the posterior nasal cavity with surgical changes of sphenoid sinus. Red arrow shows the Rathke's cyst location The image shows the sphenoid sinus anatomy and posterior nasal cavity prior to revision surgery, demonstrating the baseline endoscopic appearance before marsupialization of the recurrent Rathke's cleft cyst

A 76-year-old man with type 2 diabetes mellitus, history of meningeal tuberculosis, panhypopituitarism, and multiple failed Rathke's cleft cyst resection surgeries (two transnasal endoscopic, one transorbital craniotomy in 2021) presented with progressive visual decline. Ophthalmological evaluation revealed right temporal hemianopsia and reduced visual acuity (Figure 1).

**Figure 2:**
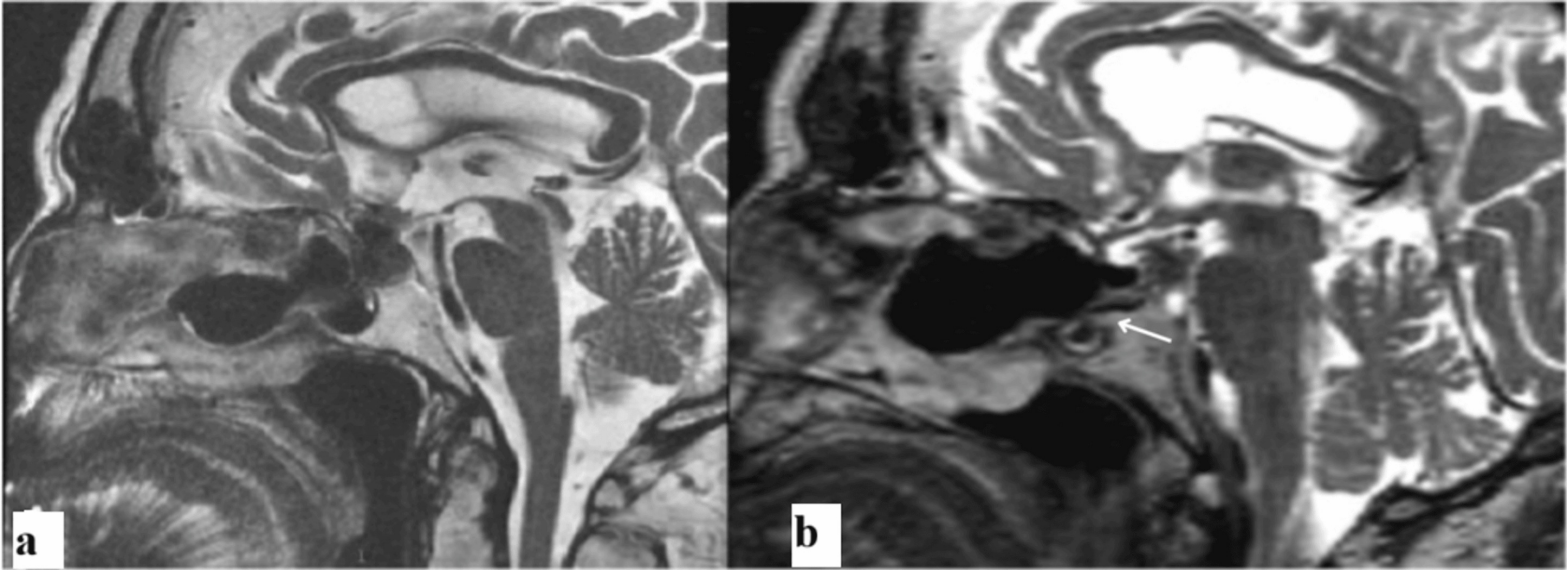
Magnetic resonance imaging T2-weighted sagittal sequences comparing pre- and postoperative findings (a) Presurgical image showing recurrent Rathke's cleft cyst with suprasellar extension and compression of the optic chiasm. (b) Eight-month postoperative image, demonstrating successful decompression of the optic chiasm with stable postoperative changes and no evidence of cyst reaccumulation. Communication between the Rathke's cleft cyst floor and the nasal cavity is shown using a white arrow

Magnetic resonance image confirmed recurrence of a sellar-suprasellar lesion compressing the optic chiasm (Figure 2a).

**Figure 3:**
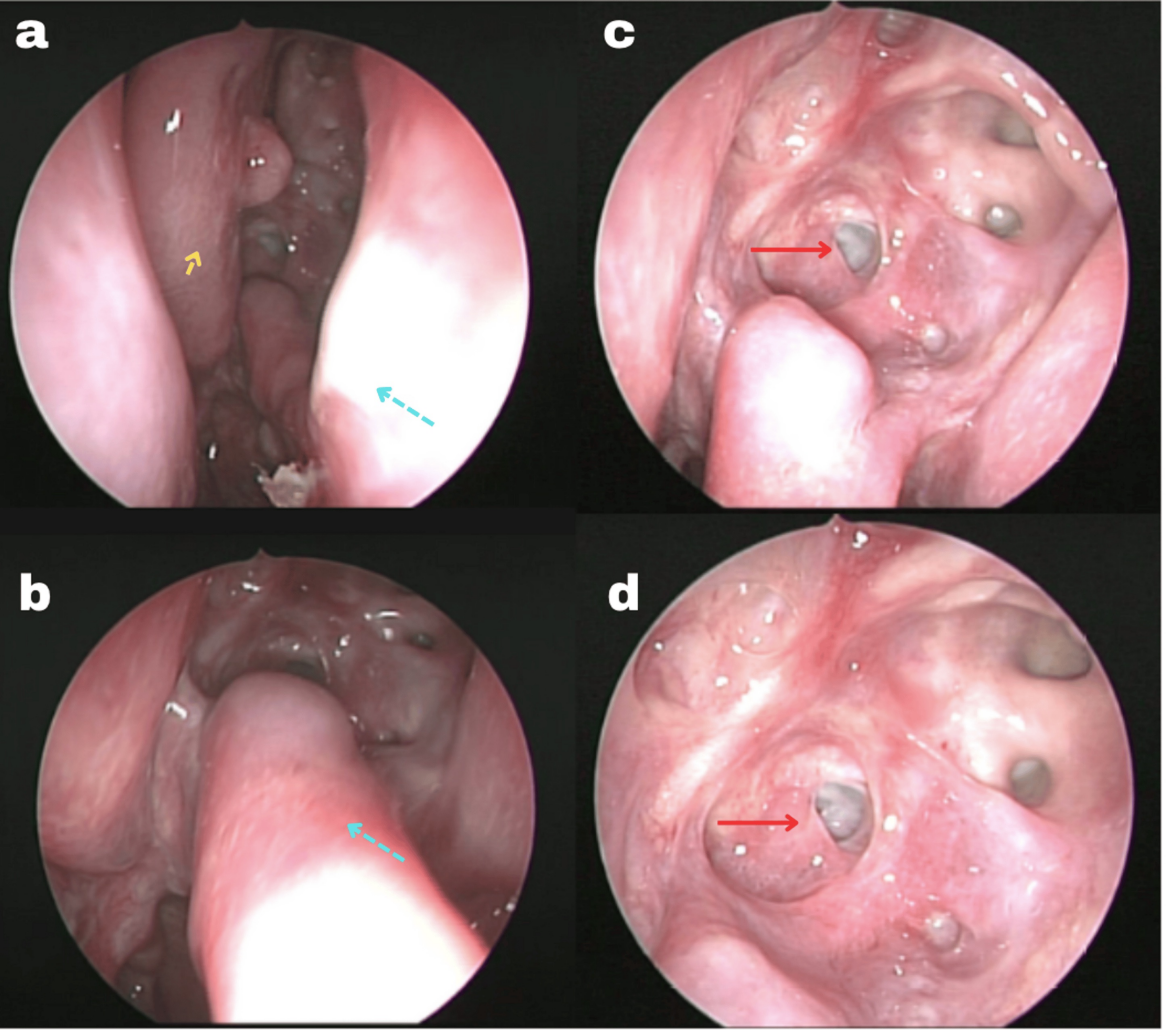
Postoperative endonasal endoscopy sequence demonstrating successful marsupialization. (a) Anterior endonasal view showing the septum (blue arrow) and middle turbinate (yellow arrow). (b) Anterior endonasal view of the septum (blue arrow) with posterior septectomy changes. (c) Posterior endonasal view showing surgical changes in the sphenoid sinus with communication between the Rathke’s cleft cyst floor and the nasal cavity (red arrow). (d) Posterior endonasal view showing an enlarged view of the surgical changes in the sphenoid sinus with communication between the Rathke’s cleft cyst floor and the nasal cavity (red arrow) The image shows the established communication between the Rathke's cleft cyst floor and the nasal cavity, with the nasoseptal flap positioned over the cyst floor to maintain permanent drainage

Revision endoscopic endonasal surgery was performed, including enlargement of the sphenoidotomy to a type III sphenoidotomy, aperture of the cyst, marsupialization with eversion of the lateral dura membrane edges toward the nasal cavity, and incision of the medial portion of the nasoseptal flap harvested in a prior surgery to communicate the sphenoid region and the nasal cavity, placing it on the cyst floor. Surgery was completed without complications. Postoperatively, the patient remained neurologically stable, continued hormonal replacement for panhypopituitarism, and had no infectious or hemodynamic events. He was discharged after stabilization and followed clinically and radiologically. At eight months, marsupialization remained functional without recurrence (Figure 3).

Ophthalmological evaluation showed improvement of visual acuity and complete resolution of hemianopsia. Magnetic resonance image confirmed stable postoperative changes without evidence of cyst reaccumulation (Figure 2b).

The patient experienced substantial relief following marsupialization, as his visual impairments had significantly disrupted his daily activities before the surgical intervention. The progressive deterioration of his vision had rendered reading and facial recognition challenging, thereby inducing anxiety regarding his quality of life. He expressed appreciation for the comprehensive explanation of the surgical procedure and found reassurance in its minimally invasive approach. At the eight-month follow-up, he reported high satisfaction with the improvement in his vision and was pleased to have resumed his normal activities without any restrictions.

## Discussion

This case demonstrates the successful application of endoscopic transnasal marsupialization with nasoseptal flap reconstruction in a particularly challenging recurrent Rathke's cleft cyst. Our patient's excellent functional outcomes at eight-month follow-up, complete resolution of hemianopsia, and improved visual acuity without recurrence underscore the efficacy of this approach in complex revision scenarios.

The management of recurrent Rathke's cleft cysts represents a significant clinical challenge. Published recurrence rates following initial surgical intervention range widely from 10% to 48%, with a recent systematic review reporting an overall radiological recurrence rate of 19.8% over a mean follow-up of 50.4 months [7,8]. Our patient, having undergone three prior failed surgeries (two transnasal endoscopic and one transorbital craniotomy), exemplifies the subset of patients who develop refractory cyst recurrence despite conventional surgical approaches.

The marsupialization technique employed in this case aligns with emerging evidence supporting the maintenance of an open sellar floor to reduce recurrence risk. A 2025 meta-analysis by Dolovac et al. demonstrated that cyst fenestration, while maintaining an open sellar floor (marsupialization into the sphenoid sinus), is associated with significantly lower recurrence rates at over four-year follow-up compared with reconstruction with sellar floor closure [9]. In their study of 52 patients undergoing marsupialization, Kuan et al. reported a recurrence rate of only 10% at a mean follow-up of 20 months, supporting the durability of this approach [10]. Similarly, Solari et al. documented favorable outcomes in 29 patients treated with an endoscopic approach over a mean follow-up of 60 months [11].

The incorporation of a nasoseptal flap in our technique represents a critical modification for preventing restenosis and maintaining long-term patency. Multiple studies have demonstrated the value of this adjunct: Eide et al. found that nasoseptal flap placement reduced recurrence requiring revision surgery from 50% to less than 9% in their comparative cohort [12,13]. Joshua et al. reported no recurrences in 10 patients treated with a modified nasoseptal flap technique at 36-month median follow-up, with all patients maintaining patent fenestration on endoscopic examination [14]. The nasoseptal flap provides a vascularized epithelial lining that promotes reepithelialization of the marsupialized cavity while preventing cicatricial closure, mechanisms that likely contributed to our patient's sustained drainage and absence of recurrence.

Visual outcomes in our case merit particular emphasis. The complete resolution of right temporal hemianopsia and improvement in visual acuity represent optimal functional recovery. In a large series of 75 patients by Nakase et al., 60% presented with vision impairment preoperatively, and maintaining the fenestration open resulted in superior visual outcomes [15]. The literature consistently demonstrates that visual recovery correlates with early surgical intervention and the degree of decompression achieved. Our patient's advanced age (76 years) and prolonged visual impairment prior to revision surgery make the complete visual field recovery particularly noteworthy, as older age has been identified as a risk factor for persistent visual impairment (OR = 1.06 per year) [1].

The challenge of recurrent cysts after multiple prior surgeries cannot be overstated. Each subsequent operation increases technical difficulty due to scarring, altered anatomy, and prior manipulation of delicate neurovascular structures. In such cases, marsupialization offers distinct advantages over attempted gross total resection: it minimizes manipulation of the pituitary stalk and surrounding structures, reducing risk of new endocrinopathy; avoids extensive dissection that increases risk of CSF leak; and establishes permanent drainage that accommodates potential cyst reaccumulation. This is particularly relevant given that Millesi et al. found that complete cyst wall resection significantly increased the rate of postoperative pituitary dysfunction (50%) compared with simple fenestration (3%) [16].

Our patient's complex medical history, including type 2 diabetes mellitus, prior meningeal tuberculosis, and panhypopituitarism, presented additional considerations for surgical planning. The marsupialization approach allowed for effective decompression while minimizing additional endocrine morbidity, a crucial consideration in a patient already requiring comprehensive hormonal replacement therapy. The absence of complications, including CSF leak, infection, or worsening pituitary function, reflects the safety profile of this technique even in medically complex patients.

The role of reconstruction technique in preventing recurrence has been a subject of considerable debate. While early microsurgical series often employed sellar floor reconstruction with fat grafting, more recent evidence suggests that maintaining an open communication between the cyst cavity and sphenoid sinus reduces recurrence rates. A 2021 retrospective analysis found that reconstruction of the sellar floor was associated with a 41% recurrence rate, compared with 17% in non-reconstructed cases [8]. Our approach, utilizing the nasoseptal flap not to seal the defect but rather to line the marsupialized opening, represents an evolution in reconstruction philosophy, promoting healing and epithelialization while preserving permanent drainage.

Long-term follow-up remains essential, as some authors have reported late recurrences occurring 5-10 years postoperatively. However, our patient's eight-month imaging demonstrating stable postoperative changes without cyst reaccumulation, combined with functional marsupialization on endoscopic examination, provides early evidence of successful treatment. Extended surveillance will be necessary to confirm durability, though the maintained anatomic communication with the nasal cavity provides a theoretical advantage for long-term cyst drainage.

From a technical perspective, several surgical nuances contributed to success in this case. The enlargement of the prior sphenoidotomy to type III in this last surgery ensured adequate access and visualization. Eversion of the dural membrane edges toward the nasal cavity created a stable epithelialized tract. The strategic incision of the medial portion of the nasoseptal flap to maintain communication while providing structural support represented a modification that balanced drainage with reconstruction. These technical details, while seemingly minor, collectively contribute to the durability of marsupialization.

The patient's subjective experience deserves mention, as it underscores the profound impact of successful surgical intervention on quality of life. His progression from debilitating visual impairment that compromised reading and facial recognition to complete functional recovery illustrates the therapeutic potential of appropriately selected revision surgery. This improvement in activities of daily living represents a meaningful outcome beyond radiological success.

While our single case cannot establish definitive superiority of one technique over another, it contributes to the growing body of evidence supporting endoscopic transnasal marsupialization with nasoseptal flap reconstruction as an effective strategy for complex recurrent Rathke's cleft cysts. The technique is particularly applicable to patients who have failed conventional approaches, offering a minimally invasive option that prioritizes functional outcomes while reducing recurrence risk. Future prospective studies with larger cohorts and extended follow-up will be valuable to further define optimal patient selection criteria and refine surgical technique.

## Conclusions

Endoscopic transnasal marsupialization communicating the cyst cavity to the nasal cavity represents a safe and effective therapeutic option for recurrent Rathke’s cleft cysts, particularly in patients with multiple failed interventions. By providing permanent drainage and minimizing manipulation of delicate structures, it may reduce recurrence and improve functional outcomes. While larger series are required to confirm long-term efficacy, this technique is supported as a valuable, minimally invasive strategy in complex or refractory Rathke's cleft cysts.
